# A rare case of an immunocompetent patient with isolated pulmonary mucormycosis

**DOI:** 10.1016/j.idcr.2023.e01726

**Published:** 2023-02-28

**Authors:** Hamidreza Rouientan, Abolfazl Gilani, Roham Sarmadian, Mohammad Rezaei zadeh Rukerd

**Affiliations:** a Urology and Nephrology Research Center, Department of Urology, Shahid Labbafinejad Medical Center, Shahid Beheshti University of Medical Sciences, Tehran, Islamic Republic of Iran; b Department of Pediatric Surgery, Tehran University of Medical Sciences, Tehran, Islamic Republic of Iran; c Infectious Diseases Reseach Center, Arak University of Medical Sciences, Arak, Islamic Republic of Iran; d Gastroentrology and Hepatology Research Center, Institute of Basic and Clinical Physiology Sciences, Kerman University of Medical Sciences, Kerman, Islamic Republic of Iran

**Keywords:** Pulmonary mucormycosis, Pneumonia, Immunocompetent, Case report

## Abstract

As one of the most lethal infections caused by fungi, pulmonary mucormycosis usually affects patients who are immunocompromised. Isolated pulmonary mucormycosis in immunocompetent patients is very rare.This report describes a 65-year-old immunocompetent man, who was first treated with antibiotics after being diagnosed with a lung infection, then after the lack of response to treatment, he was diagnosed with mucormycosis in further investigations. Finally, the patient died due to a lack of response to treatment. Despite its rarity, it is important to consider other differential diagnoses such as pulmonary mucormycosis if the pneumonic process does not respond to medical treatment.

## Introduction

The term mucormycosis refers to a group of potentially life-threatening fungi that affect several different parts of the body [1], [2]. It usually occurs in immunocompromised patients suffering from hematological malignancy, or diabetes mellitus, or patients receiving long-term immunosuppressive therapy following a hematological stem cell transplant or solid organ transplant [1].

In the absence of any of the risk factors listed above, the presence of pulmonary mucormycosis is extremely rare [3]. Due to its nonspecific presentation, pulmonary mucormycosis is easily misdiagnosed, especially in immunocompetent patients, which could result in serious consequences. Since its incidence has increased significantly in recent decades, it poses a serious threat to the health of humans [4].

The present study describes a case of isolated pulmonary mucormycosis in a previously healthy adult man. This study aims to help clinicians identify pulmonary mucormycosis as early as possible, especially in immunocompetent patients, to improve therapeutic efficacy and prognosis.

## Case presentation

A 65-year-old man presented to an outpatient clinic with complaints of productive cough (without blood streaks in sputum) and chest pain lasting four weeks. He had a complaint of bilateral chest pain that was aggravated by deep breathing. His past medical history included hypertension, which was controlled by a daily dose of 50 mg of hydrochlorothiazide. His symptoms included fever, weight loss, and progressive myalgia. The patient was a lifelong nonsmoker without known sick contact and recent travel. Vital signs were stable. Apart from bilateral rales in his lower lungs, his physical examination was otherwise unremarkable.

**Fig. 1:**
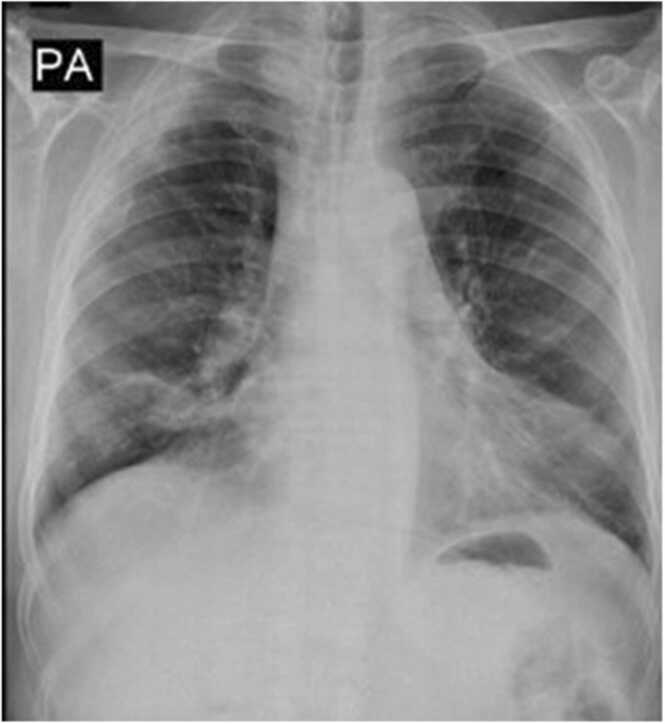
Chest X-ray posterior-anterior view showing bilateral nonhomogeneous opacities in the lower zone.

Relevant laboratory findings were as follows: white blood cell: 12.9 × 10^9/L (reference interval: 4–10 ×10^9/L); neutrophil%: 86.9 % (reference interval: 40.0–75.0 %); lymphocyte%: 10.8 % (reference interval: 20.0–50.0 %); C-reactive protein = 17 mg/L, erythrocyte sedimentation rate = 56 mm/h. Chest radiograph showed bilateral nonhomogeneous opacity in the lower zone (Fig. 1). The result of the polymerase chain reaction (PCR) for SARS-CoV-2 was reported as negative. On the same day, the patient was discharged in good general condition with oral antibiotics (625 mg co-amoxiclav, three times a day for ten days).

**Fig. 2:**
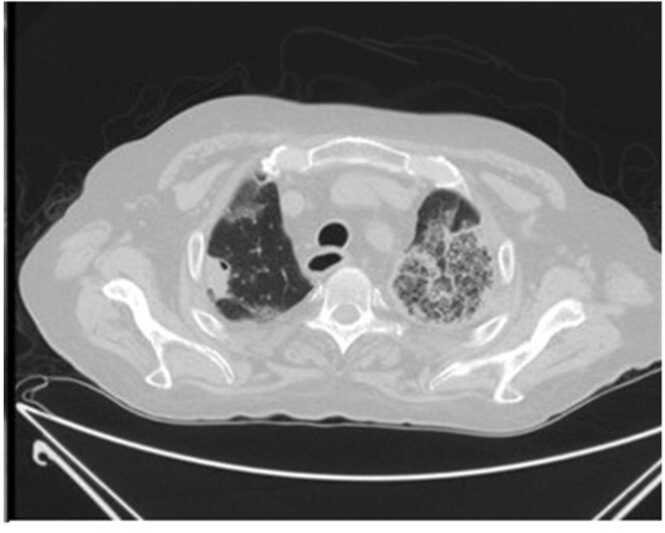
Chest CT scan showing bilateral ground glass opacities and consolidations in lower lobes of the lungs.

He returned after 17 days with complaints of dyspnea and fever. There were no significant findings in the initial laboratory test and physical examination in this admission, except for an oral temperature of 38.3 ℃ and a white blood cell count of 13.5 × 10^9/L (reference interval: 4.0–10.0 × 10^9). SARS-CoV-2 PCR was performed again, and the result was negative. Video bronchoscopy showed a bilateral purulent secretion with bronchoalveolar lavage negative for mycobacterium. Electrocardiography and echocardiography were both normal during the cardiology consultation. After 2 days of intravenous antibiotics administration, the patient was discharged in good general condition. On the 44th day following the onset of his initial symptoms, he returned with dyspnea and right leg edema. During the physical examination, bilaterally decreased lung sounds and decreased oxygen saturation were observed. Abnormal paraclinical tests were as follows: white blood cells 15.8 × 10^9/L (reference interval: 4.0–10.0 × 109) and deep vein thrombosis (DVT) in the right leg on ultrasound. The electrocardiogram, urine test, liver function, electrolyte test, and renal function were all normal. Fig. 2 shows the lung Computed tomography (CT) scan taken on this admission. The chest CT scan revealed pulmonary infection without a pulmonary embolism. Accordingly, treatment for severe community-acquired pneumonia including ceftriaxone ampule 1 gr every 12 h, and levofloxacin ampule 500 mg daily, as well as DVT treatment including apixaban 10 mg twice daily (BID) for seven days and then 5 mg twice daily were continued.

On day 51, after no significant improvement in the patient's chief complaint, a lung Computed tomography, bronchoscopy, and transbronchial biopsy were performed.

The bronchoscopy revealed purulent secretions, and the lung Computed tomography revealed necrotizing pneumonitis. Therefore, intravenous antibiotics consisting of vancomycin ampule 1 g immediately and 1 g every 12 h, meropenem ampule 2 g immediately and 1 g every 8 h, and levofloxacin ampule 500 mg daily were administered.

**Fig. 3:**
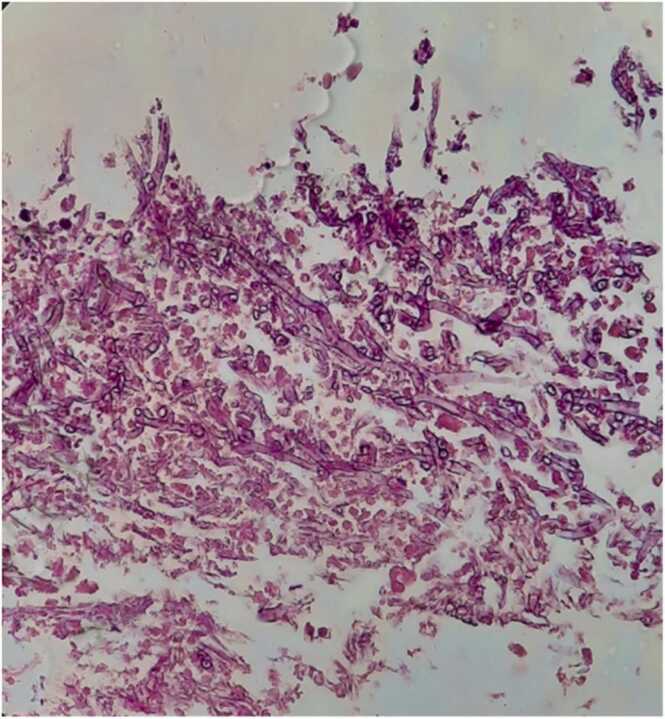
Lung biopsy specimen showing colonies of broad, right-angled branching, non-septate hyphae (40 × magnification).

On day 54, a transbronchial biopsy was performed, and while there was no growth in the culture, histology revealed mucormycosis (Fig. 3). Therefore, treatment with linezolid 500 mg two times a day, colomycin three times a day, and amphotericin B liposomal 300 mg daily was initiated.

**Fig. 4:**
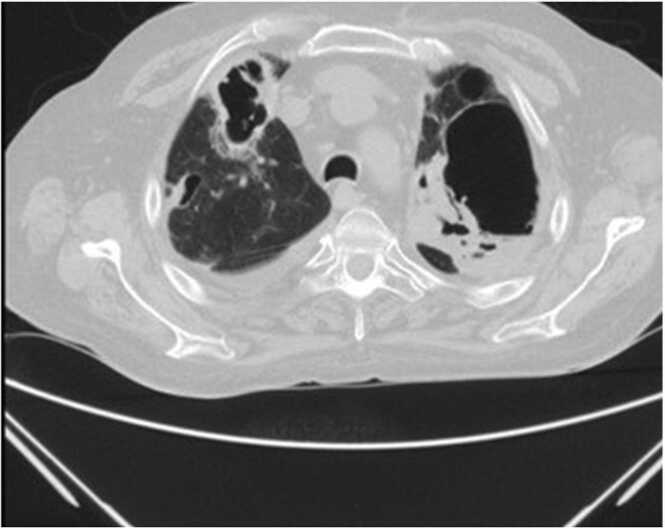
Chest CT scan showing progression of ground glass opacities into consolidation, and large cavitary lesions in bilateral lung fields.

The patient's condition deteriorated during the hospitalization. The lung Computed tomography after 19 days of treatment for mucormycosis is shown in Fig. 4.

Twenty-five days after the initiation of the specific treatment for mucormycosis, the patient did not respond to the therapy and succumbed to disseminated intravascular coagulation and respiratory failure which eventually led to death.

## Discussion

Mucormycosis is an acute, progressive, and fatal disease that can affect people of almost all ages. Immunosuppression is considered to be the most important risk factor for mucormycosis [1]. Fungal growth produces an abundance of airborne spores that are constantly inhaled by humans. Despite constant exposure, infections are rare because the immune system has the ability to effectively neutralize these spores. Clinically, there are only a few cases of pulmonary mucormycosis in immunocompetent individuals. According to a systematic review conducted by He et al. between 2010 and 2020, only 16 cases of isolated pulmonary mucormycosis in immunocompetent patients have been reported [3]. In their research, nearly all patients were diagnosed either through bronchoalveolar lavage culture (50 %) or histopathologic assessment of biopsy (42 %); the remaining case was verified via autopsy.Overall, about 43 % of patients with confirmed fungal infection died despite receiving antifungal drugs. Our case was also an immunocompetent patient identified with pulmonary mucormycosis following a transbronchial lung biopsy, and antifungal treatment was initiated. In the end, though, he did not respond to the treatment and expired.

The history of exposure to mucor spores from decaying food, soil, and animal excrement may serve as a diagnostic basis for pulmonary mucormycosis [5]. In the case we described, the patient was a 65-year-old farmer and rancher.

In terms of diagnosing the disease at an early stage, conventional diagnostic techniques, such as clinical diagnosis, serology, histopathology, and radiology, have limitations. Therefore, advanced diagnostic tools such as nucleic acid diagnostics, advanced serological tests, PCR (pan-Mucorale test), and multiplex PCR are necessary. The use of these techniques allows clinicians to detect this invasive fungus at an incipient stage, preventing adverse outcomes [6]. In our case, mucormycosis was not initially considered a differential diagnosis, and pan-mucorale PCR was not requested, because the patient had no underlying disease. The transbronchial biopsy and subsequent histopathological assessment led to the diagnosis.

Lobectomies have been shown to be successful in curing individuals with early-stage lung infection [7], [8]. Unfortunately, many patients present with severe involvement that cannot be resected, and/or severe thrombocytopenia that makes surgery impossible. In these cases, antifungals should be given as soon as possible, and efforts should be made to improve immune function and treat any underlying medical issues that may be contributing to the fungal infection [9]. Our patient was given a definitive diagnosis when he had severe lung involvement and surgery could have little effect on enhancing his prognosis. Thus, he did not undergo surgery and antifungal medication was administered; nevertheless, it had little effect on the disease's improvement, and the patient died.

## Conclusion

In conclusion, pulmonary mucormycosis in immunocompetent patients is rare, which indicates the importance of being alert to potential mucormycosis infections in immunocompetent patients. Due to the high mortality rate of this disease, early diagnosis and treatment are vital to the prognosis of patients.

## CRediT authorship contribution statement

Conceptualization: Roham Sarmadian. Methodology: –. Validation: Abolfazl Gilani. Formal analysis: –. Investigation: Abolfazl Gilani, Roham Sarmadian. Resources: Abolfazl Gilani. Data curation: –. Writing – original draft: Hamidreza Rouientan, Mohammad Rezai zadeh Rukerd. Writing – review & editing: Roham Sarmadian, Mohammad Rezai zadeh Rukerd. Visualization: Hamidreza Rouientan. Supervision: Roham Sarmadian. Project administration: Abolfazl Gilani. Funding acquisition: –.

## Ethical approval

Ethical issues (including plagiarism, data fabrication, and double publication) have been completely observed by the authors. The patient’s legal guardians (his wife and children) gave written informed consent to publish as a case report. Our institution does not require ethical approval for reporting individual cases or case series.

## Consent

This case report was conducted in accordance with the World Medical Association Declaration of Helsinki. The patient’s legal guardians (his wife and children) have given us informed consent for publication as a case report.

## Funding

This research did not receive any funding.

## Competing Interest

The authors disclose no competing interest.
